# Microstimulation Is a Promising Approach in Achieving Better Lead Placement in Subthalamic Nucleus Deep Brain Stimulation Surgery

**DOI:** 10.3389/fneur.2021.683532

**Published:** 2021-09-22

**Authors:** Lin Shi, Shiying Fan, Tianshuo Yuan, Huaying Fang, Jie Zheng, Zunyu Xiao, Yu Diao, Guanyu Zhu, Quan Zhang, Huanguang Liu, Hua Zhang, Fangang Meng, Jianguo Zhang, Anchao Yang

**Affiliations:** ^1^Department of Neurosurgery, Beijing Tiantan Hospital, Capital Medical University, Beijing, China; ^2^Department of Functional Neurosurgery, Beijing Neurosurgical Institute, Beijing, China; ^3^Beijing Advanced Innovation Center for Imaging Theory and Technology, Capital Normal University, Beijing, China; ^4^Academy for Multidisciplinary Studies, Capital Normal University, Beijing, China; ^5^Department of Ophthalmology, Children's Hospital, Harvard Medical School, Boston, MA, United States; ^6^Molecular Imaging Research Center, Harbin Medical University, Harbin, China

**Keywords:** deep brain stimulation, microstimulation, microelectrode recording, optimal lead placement, substantia nigra, subthalamic nucleus

## Abstract

**Background:** The successful application of subthalamic nucleus (STN) deep brain stimulation (DBS) surgery relies mostly on optimal lead placement, whereas the major challenge is how to precisely localize STN. Microstimulation, which can induce differentiating inhibitory responses between STN and substantia nigra pars reticulata (SNr) near the ventral border of STN, has indicated a great potential of breaking through this barrier.

**Objective:** This study aims to investigate the feasibility of localizing the boundary between STN and SNr (SSB) using microstimulation and promote better lead placement.

**Methods:** We recorded neurophysiological data from 41 patients undergoing STN-DBS surgery with microstimulation in our hospital. Trajectories with typical STN signal were included. Microstimulation was applied near the bottom of STN to determine SSB, which was validated by the imaging reconstruction of DBS leads.

**Results:** In most trajectories with microstimulation (84.4%), neuronal firing in STN could not be inhibited by microstimulation, whereas in SNr long inhibition was observed following microstimulation. The success rate of localizing SSB was significantly higher in trajectories with microstimulation than those without. Moreover, results from imaging reconstruction and intraoperative neurological assessments demonstrated better lead location and higher therapeutic effectiveness in trajectories with microstimulation and accurately identified SSB.

**Conclusion:** Microstimulation on microelectrode recording is an effective approach to localize the SSB. Our data provide clinical evidence that microstimulation can be routinely employed to achieve better lead placement.

## Introduction

Deep brain stimulation (DBS) of the subthalamic nucleus (STN) is a well-accepted therapeutic approach for controlling the motor symptoms of Parkinson's disease (PD) (1, 2). The efficacy of STN-DBS heavily relies on the influence by the appropriate placement of DBS electrodes relative to STN (3, 4). Previous studies suggested an optimal lead placement trajectory that travels through the entire dorsolateral portion of STN, with the tip of the distal contacts placed near the deep boundary of STN (3, 5, 6), which will enhance clinical outcomes (1, 2, 7, 8). However, the major challenge of this optimal lead placement is how to precisely localize STN, especially the ventral boundary (6, 9) of STN that is an almond-shaped nucleus embedded deeply in between the diencephalon and the midbrain and surrounded by multiple brain structures. Beneath the STN lies the substantia nigra (SN), which can be divided histologically into the dorsolateral substantia nigra pars reticulata (SNr) and the ventromedial substantia nigra pars compacta (6, 9). The ventral boundary of the STN is very close to SNr (6, 10), and the gap between them is only 0.5–1 mm or even smaller (6, 11). Due to insufficient imaging resolution and low signal-to-noise ratio (9, 11), conventional magnetic resonance imaging (MRI) strategies often fail in distinguishing STN from SNr.

Electrophysiological recording technique is often used to help distinguish the anatomical borders between the STN and SNr. Compared to the STN, SNr tends to have a neuronal activity featured by a higher and more regular firing frequency with a lower background noise level (3, 6, 10). Besides this, the appearance of the small inactive area (silent area) at the boundary between the STN and SNr (SSB) can also help identify the ventral border of the STN (5, 12). However, sometimes the inactive area is too small, if not missing, to be captured or noticed by intermittent electrophysiological recording, leading to a difficulty in recognizing the beginning of SNr (6, 11). In such cases, neurosurgeons often have to determine the implantation depth based on personal experiences along with the trial stimulation effects, which in many cases brings uncertainties to the efficacy of DBS.

Microstimulation is a train of electrical stimulation pulses with small electrical current delivered *via* the tip of a microelectrode, which is often used to pre-evaluate the stimulation effects of macroelectrodes (13, 14). A previous study has suggested that microstimulation can trigger different responses in STN and SNr (15). To further test this potential of microstimulation, we conducted this study and evaluated the feasibility of microstimulation in distinguishing the STN from SNr on patients who received STN-DBS surgery. Our data demonstrates that microstimulation is a promising tool in detecting SSB and guiding the placement of DBS leads.

## Methods

### Patients

The data analyzed in the study were recorded from patients who received microstimulation to determine SSB during unilateral or bilateral STN-DBS surgery as a treatment for PD or dystonia at Beijing Tiantan Hospital between October 2019 and December 2020. Their intraoperative electrophysiological recording data were reviewed so that trajectories with poor neural signal or trajectories in which electrodes deviated significantly from STN, as shown in postoperative reconstruction (see below), were excluded. The study was performed in accordance with the Declaration of Helsinki, and all procedures were approved by the Institutional Review Board of our hospital (permission no. KY2019-097-02). Informed written consents were acquired from all patients. This case series has been reported in line with the PROCESS Guideline (16).

### Surgical Procedures and Microstimulation

**Figure 1:**
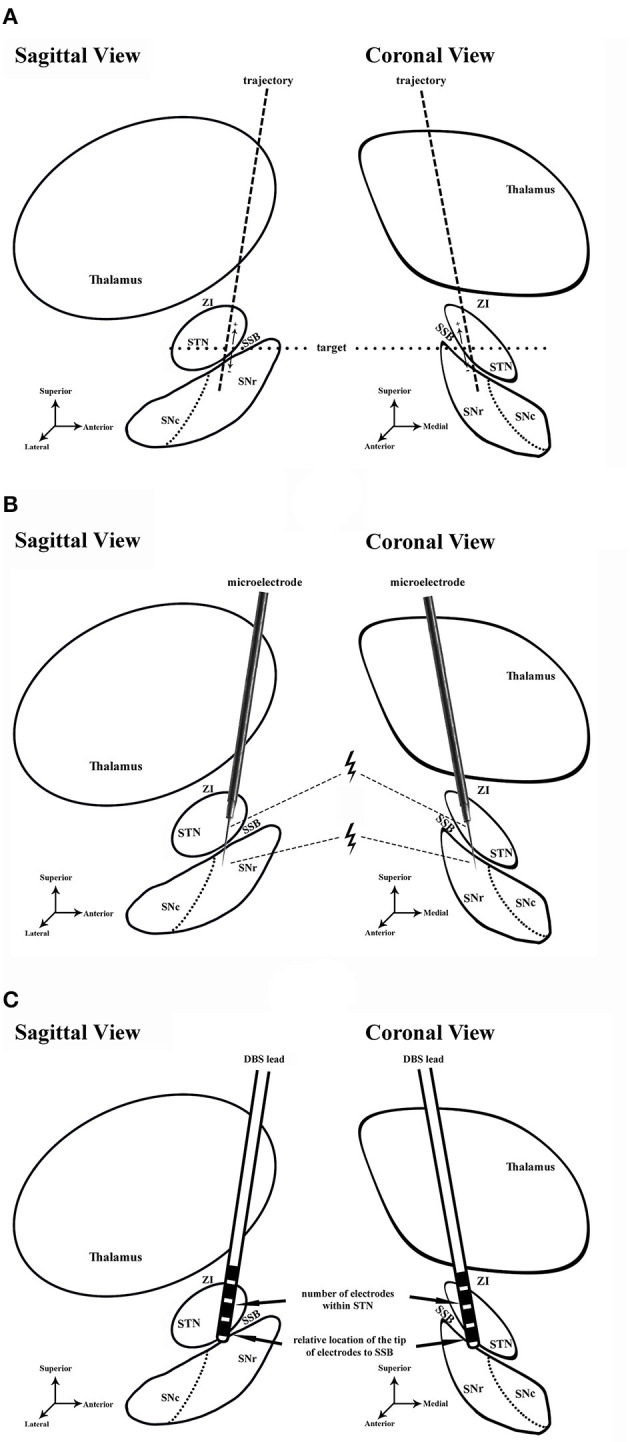
Schematic diagram of the study. **(A)** Preoperative planning in sagittal and coronal views. Dash line, trajectory of lead placement. Dot line, the level of target. **(B)** Microelectrode recording and microstimulation during the DBS surgery. Microstimulation was conducted in the ventral STN and dorsal SNr. Thunder symbol, microstimulation near the exit of STN and entrance of SNr. **(C)** Assessment of lead placement. The number of electrodes in STN (arrow) and the relative location of the electrode tip to SSB (arrow) were assessed to evaluate the lead placement. STN, subthalamic nucleus; ZI, zona incerta; SNr, substantia nigra pars reticulata; SNc, substantia nigra pars compacta; SSB, boundary between subthalamic nucleus and substantia nigra; DBS, deep brain stimulation.

**Figure 2:**
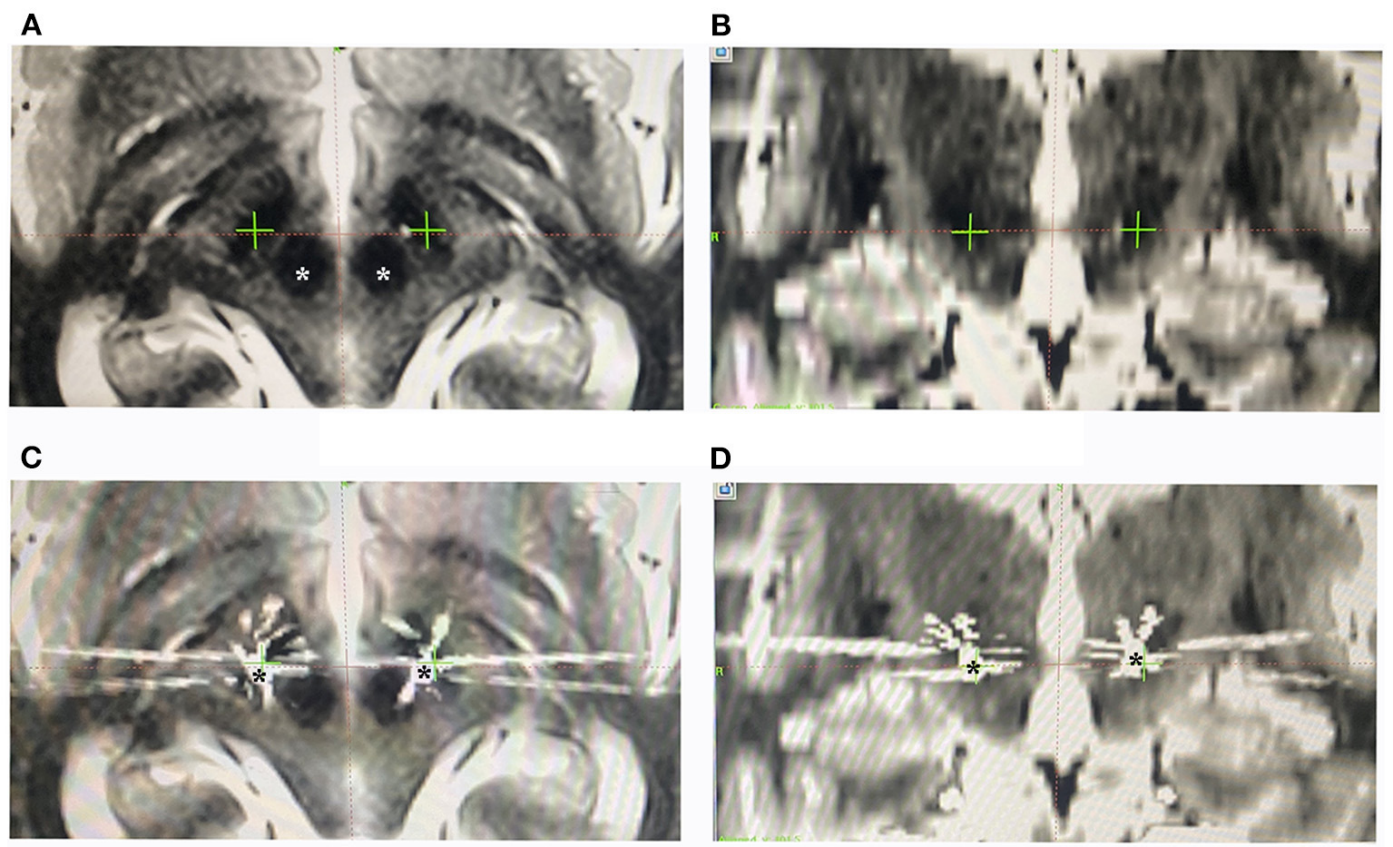
Preoperative planning for lead implantation and postoperative verification of the lead location. **(A,B)**, Preoperative planning on MRI images using the SurgiPlan software, with the targets (green crosses) placed along the anterior edge of the red nucleus (white asterisk). **(C,D)** Postoperative verification of the lead location (black asterisk) using the SurgiPlan software to overlap postoperative CT images onto preoperative MRI images.

All patients went through a routine neurological assessment conducted by movement disorder specialists, including the Unified Parkinson's Disease Rating Scale (UPDRS) for PD patients, the Unified Dystonia Rating Scale (UDRS), and multiple other scales and examinations, *etc*., to confirm the diagnosis and indication for surgery (5, 12). The preoperative brain MRI scans of patients were acquired at admission using a 3-Tesla MRI scanner (SIGNA; GE Healthcare, Waukesha, WI, USA), including 3D-T1-weighted (slice thickness: 1 mm, repetition time: 9.4 ms, echo time: 4.3 ms, spacing between slices: 0), axial T2-weighted (slice thickness: 1 mm, repetition time: 7,881 ms, echo time: 104.9 ms, spacing between slices: 0), and coronal T2-weighted (slice thickness: 1 mm, repetition time: 8,947 ms, echo time: 102 ms, spacing between slices: 0) images. The patients received a unilateral (two cases) or bilateral (39 cases) DBS surgery. The procedures of the surgery were described in detail in previous studies (12, 17, 18). Targeting and trajectory planning were conducted on an image-based preoperative planning system (Leksell SurgiPlan 10.1, Elekta Instrument, Sweden) using the direct targeting method as described before (19–21) (Figure 1A, Figures 2A,B). On each side, after incising the scalp and making a burr hole under local anesthesia, a shielded tungsten microelectrode (Neuroprobe, Alpha Omega, Israel; an average impedance of 458 ± 183 kΩ) was inserted into the brain toward the target area driven by a microdrive (Drive Headstage, Alpha Omega, Israel). Electrophysiological signals were recorded to guide the implantation of the DBS leads (Figure 1B) using a Neuro Omega system (Neuro Omega, Alpha Omega, Israel). Microelectrodes were advanced at a step-size of 0.2–0.4 mm once the entrance of the STN was identified, and when the tips of the microelectrodes were near the bottom of the STN, the step-size was adjusted to 0.1–0.2 mm. The gap between the exit of the STN and the putative entrance of SNr was considered SSB. The thickness of the SSB was annotated.

We conducted microstimulation (0.5-s train at 200 Hz in frequency, 300 μA in amplitude, and 60 μs in pulse width) at every stop near the bottom of the STN to investigate the neuronal responses using a similar approach as described in previous literature (15) (Figure 1B). At every stop, the microstimulation was repeated two to four times. The recorded signal was examined to determine whether microstimulation induced the inhibition of neuronal firing. The inhibition period was defined as the time length between the end of the microstimulation train and the first spike afterwards. If the inhibition periods were unidentifiable (lower than 50 ms) in the STN and much longer in SNr (longer than 50 ms, usually longer than 200 ms), the electrophysiological recording in this trajectory was considered concordant with the findings described in previous studies (15, 22); otherwise, it was considered discordant.

When a trajectory with satisfactory signal was obtained, a DBS lead was implanted along the same track for trial stimulation (Figure 1C). The therapeutic windows (*i.e*., the range between the minimal intensity of stimulation required to obtain meaningful clinical benefits) and the intensity of stimulation at which the first persistent side effect occurred were noted. UPDRS or UDRS assessments were performed by a neurologist. The ultimate decision on the implantation depths of DBS leads in the track was made based upon the consensus among the neurosurgeons, a neurologist, and a neurophysiologist after considering the length of STN, the possible location of SNr, the intraoperative UPDRS/UDRS assessments performed by the neurologist, side effects, and the therapeutic window. The locations of the lead tips were noted for further verification.

### Lead Reconstruction

**Figure 3:**
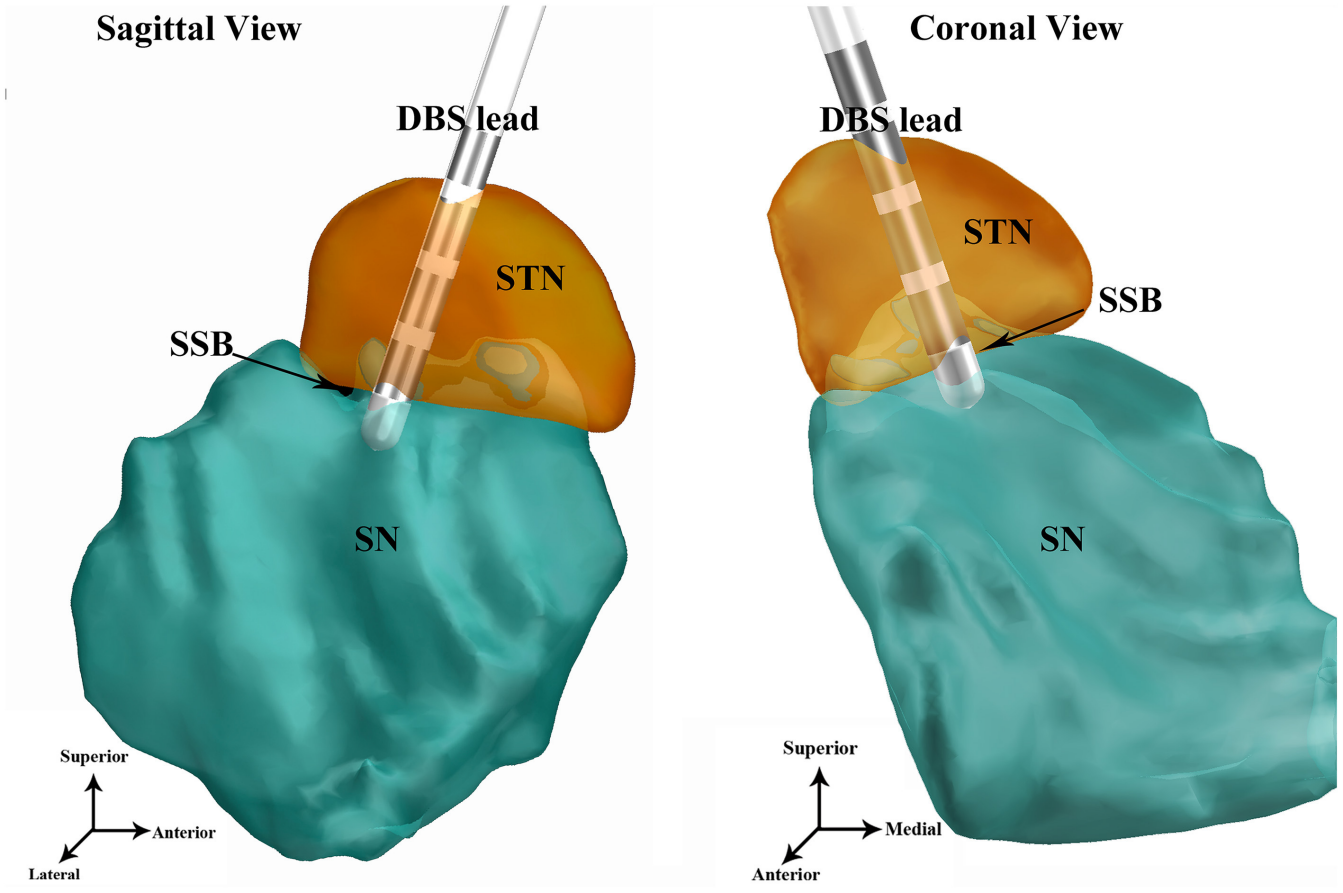
An example of DBS lead reconstruction using the software Lead DBS. The electrode tip of the DBS lead is placed in or very close to SSB. DBS, deep brain stimulation; STN, subthalamic nucleus; SN, substantia nigra; SSB, boundary between subthalamic nucleus and substantia nigra.

After surgery, a standardized postoperative computed tomography (CT) scan was conducted according to existing protocol (12). The postoperative CT was co-registered to the preoperative MRI to localize the electrodes and the tip of the contact array. A semi-automated Matlab toolbox, the Lead DBS toolbox (23), was used to visualize the leads in 3D virtual space (Figure 3). The precision of the reconstruction was verified by overlapping the postoperative CT upon the preoperative MRI (Figures 2C,D). The trajectories whose electrodes were obviously outside STN were excluded. The location of the lead was assessed according to the relative relationship between the tip of the contact array and SSB. The location of the lead was considered satisfactory if the ventral tip of the contact array was within or very close to SSB (*i*.*e*., the distance between the tip of contacts and SSB was smaller than 1 mm) as revealed by Lead DBS and the notes of microelectrode recording. The number of contacts within the STN nucleus shown in the reconstructed images was counted. A contact was counted as half if only part of the contact was inside of the STN.

### Statistical Analyses

We employed the Fisher exact test for categorical data and the *t*-test for continuous data to determine statistical significance. All statistical analyses were performed using R software (version 4.0.2) (24). The number of SSB identification was calculated for two groups (trajectories with macrostimulation *vs*. trajectories without microstimulation). The Fisher exact test was used to test the significance of proportion difference between groups with and without microstimulation. Descriptive statistics including means and standard errors were calculated. Unpaired two-tailed Student's *t*-test was used to test the significance of the mean difference between groups with identified SSB and without identified SSB.

## Results

### Data Overview

**Figure 4:**
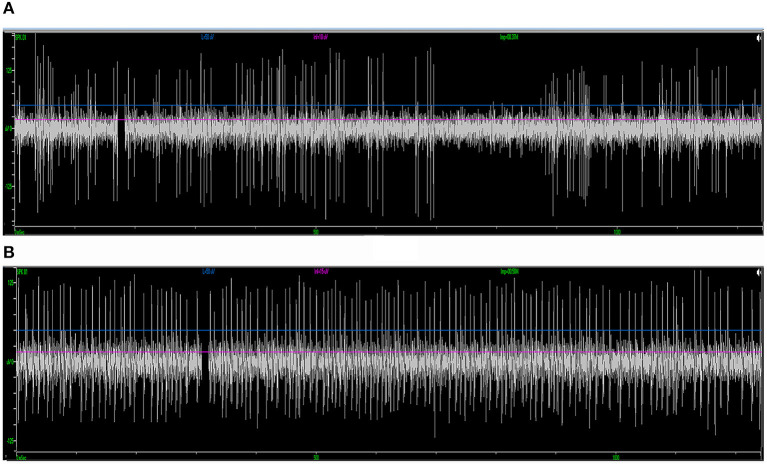
Examples of standard STN and SNr signal in intraoperative microelectrode recording. **(A)** The typical STN signal is highlighted with a higher background noise level and irregularly firing neurons. **(B)** The SNr signal is featured by a lower background noise level with neurons that spike more regularly and frequently. STN, subthalamic nucleus; SNr, substantia nigra.

Forty-one patients (16 males and 25 females; 31 PD and 10 dystonia cases in the cohort, with an average age of 58.2 ± 10.9 years) who underwent STN-DBS surgery (two unilateral and 39 bilateral) were included in the study. The average length of the disease was 8.5 ± 5.3 years. In total, 80 sessions of neural data that were recorded from microelectrodes implanted through different trajectories on these patients were analyzed. Typical STN signal was recorded in 50 trajectories (62.5%) (Figure 4A). In the rest of the 30 trajectories (37.5%), the signal was atypical inside the nucleus. In 56 trajectories (70.0%), putative SNr signal was identified at 1 mm above to 5 mm below the target, featured by a higher firing rate and a lower background compared to STN (Figure 4B). In the other 24 trajectories (30.0%), SNr signal was either not reached out for because a satisfactory coverage of STN was achieved or not identified during the microelectrode recording.

### Inhibitory Responses Induced by Microstimulation in STN and SNr

**Figure 5:**
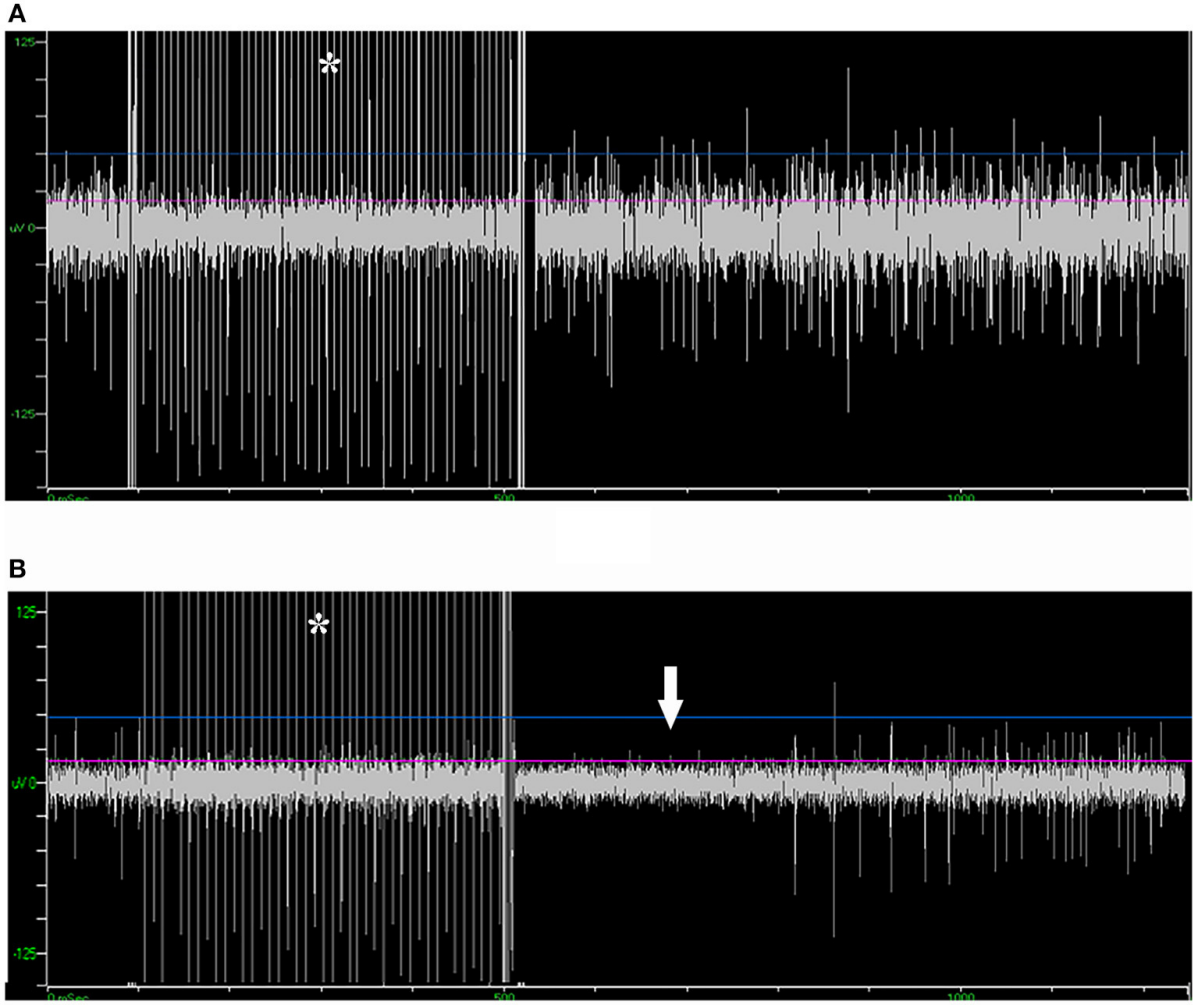
Microstimulation-induced inhibition of neuronal activity in SNr but not in STN. **(A)** Microstimulation (asterisk) in STN failed to induce inhibition in neuronal activity. **(B)** Microstimulation induced long inhibition (arrow) in SNr. STN, subthalamic nucleus; SNr, substantia nigra.

Of the 39 patients who underwent bilateral surgery, 35 of them received unilateral and five received bilateral microstimulation. In total, 155 trials of microstimulation were applied in 45 trajectories (56.3%), while in the other 35 trajectories (43.8%) microstimulation was not conducted. No patient reported any discomfort with microstimulation, and no abnormal scenario (like seizure) was induced. In 38/45 trajectories with microstimulation (84.4%), STN signal could not be inhibited by microstimulation, while SNr presented a long inhibition period following microstimulation (566 ± 217 ms, Figure 5), which was in line with the previous study (15). By contrast, in three trajectories (6.7%), mild inhibitory responses were induced near the bottom of the STN (162 ± 77 ms), and a putative SNr signal could not be inhibited in the other four trajectories (8.9%).

### Microstimulation Promotes the Identification of SSB

**Figure 6:**
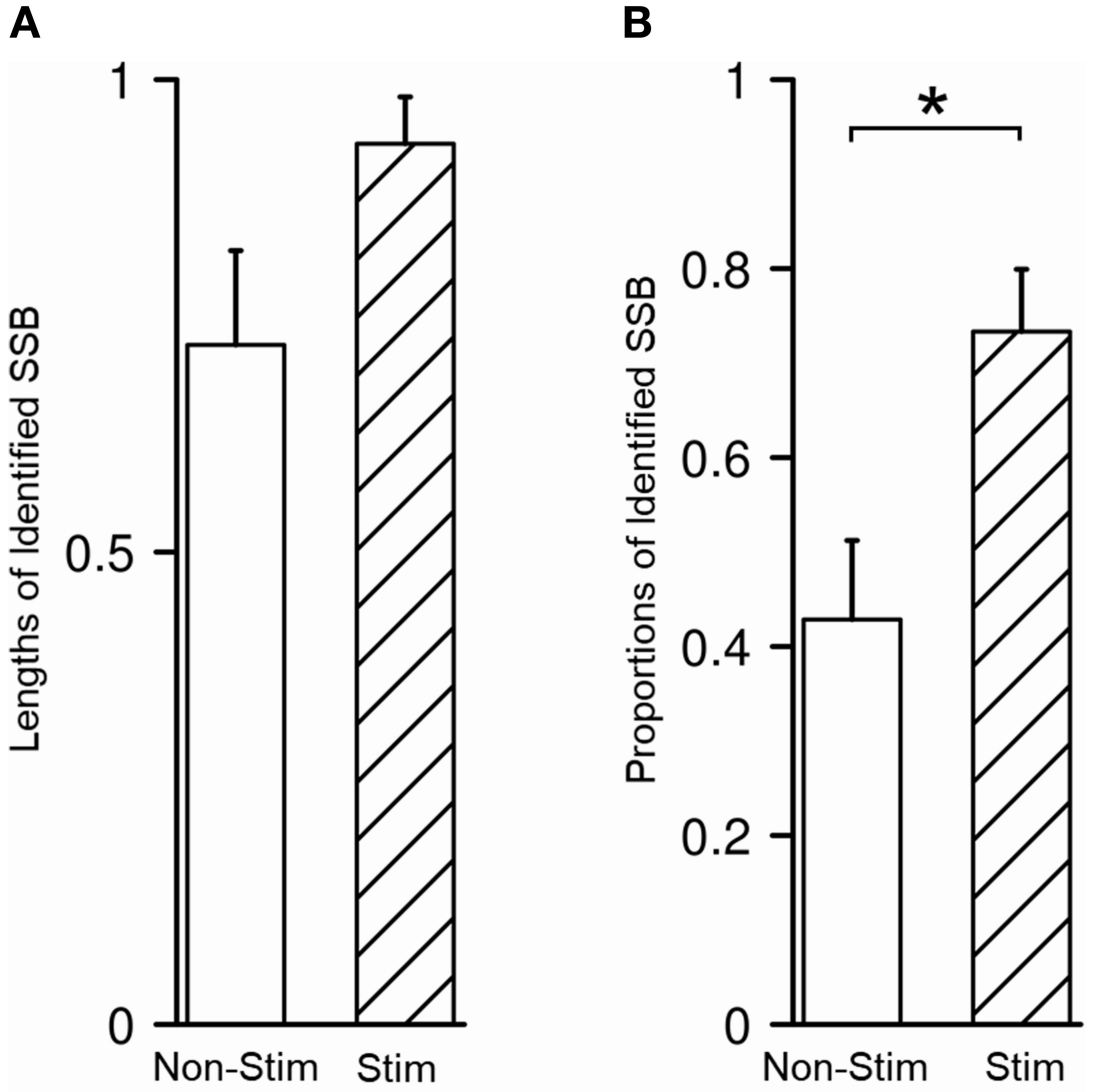
Microstimulation promoted the identification of SSB. **(A)** There was no difference between the length of SSB in trajectories with and without microstimulation. **(B)** The proportion of identified SSB was significantly higher in trajectories with microstimulation than those without. SSB, boundary between subthalamic nucleus and substantia nigra. *P < 0.05.

In our study, SSB was identified in 48/80 trajectories (60.0%), either purely by recognizing the exit of STN and the entrance of SNr in microelectrode recording or with the aid from microstimulation to test the inhibitory responses of STN and SNr. The average length of SSB was 0.87 ± 0.32 mm. No difference was detected between the length of SSB in trajectories with and without microstimulation (*P* = 0.070, Figure 6A). The identification rate of SSB was further compared between the tracks with and without microstimulation. Using microstimulation, SSB was identified in 33/45 trajectories (73.3%), which is significantly higher than those without microstimulation where SSB was found in only 15/35 trajectories (42.9%, *P* = 0.011, Figure 6B).

### Imaging Reconstruction of DBS Leads

**Figure 7:**
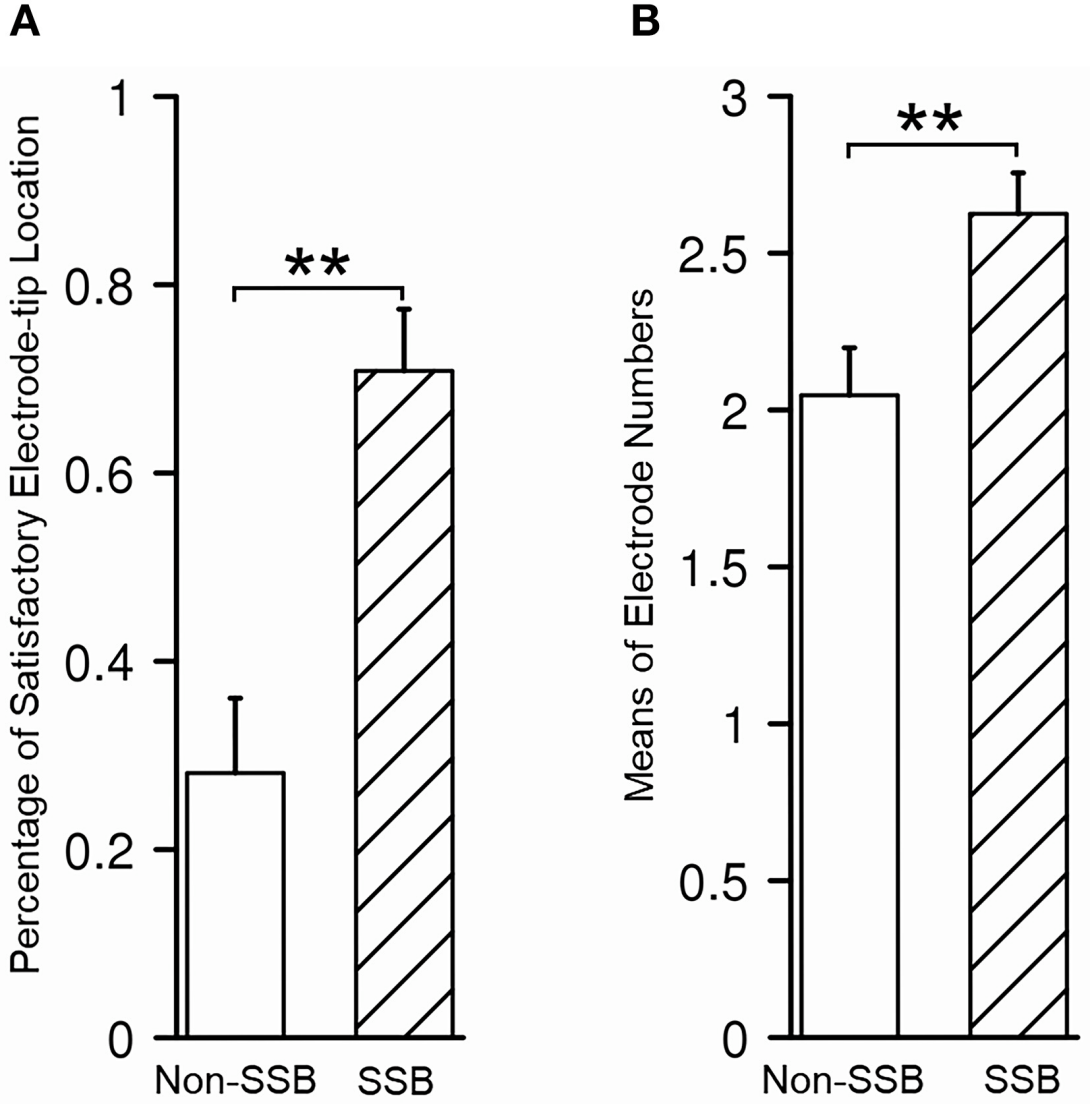
Assessment of lead placement in trajectories with identified SSB and those without. **(A)** The percentage of satisfactory relative electrode tip location was higher in trajectories with identified SSB than those without. **(B)** The mean electrode number within STN was higher in trajectories with identified SSB than those without. SSB, boundary between subthalamic nucleus and substantia nigra. **P < 0.001.

In 43/80 trajectories (53.8%), the distal tips of the contacts were placed within or very close to SSB, which is considered satisfactory, while in the other 37/80 trajectories, the distal tips of the contacts were at least 1 mm away from SSB (46.2%), which was considered unsatisfactory. In all trajectories, the mean number of contacts within STN was 2.4 ± 0.9. To determine whether the recognition of SSB might benefit the lead placement, we compared the relative location of the electrode tip and the number of contacts within STN between the trajectories whose SSB was identified and whose SSB was not identified. The percentage of a satisfying electrode tip location was 70.8% in trajectories with SSB identified and 28.1% in trajectories whose SSB was not identified (*P* = 0.0002, Figure 7A). On the other hand, the contact number within the STN in trajectories with the SSB identified was 2.6 ± 0.9, which was significantly higher than those trajectories whose SSB was not identified (2.0 ± 0.9, *P* = 0.005, Figure 7B).

### Microstimulation Contributes to Better Therapeutic Effectiveness

**Figure 8:**
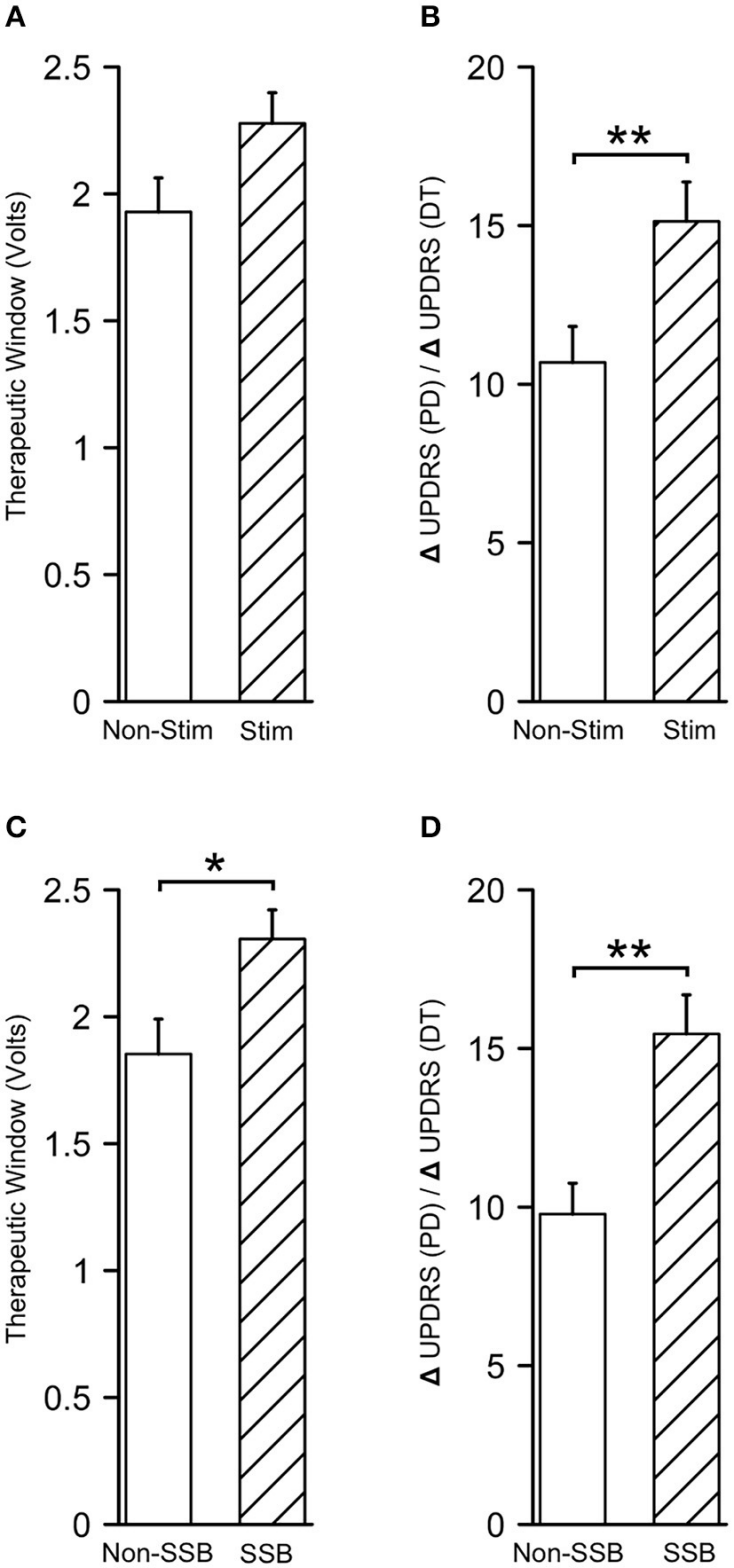
Microstimulation contributes to better therapeutic effectiveness. **(A,B)** Therapeutic windows and differences in UPDRS/UDRS scores between the preoperative and intraoperative assessments (ΔUPDRS/ΔUDRS) in tracks with and without microstimulation. **(C,D)** Therapeutic windows and ΔUPDRS/ΔUDRS scores in the SSB and non-SSB tracks. SSB, boundary between subthalamic nucleus and substantia nigra. *P < 0.05; **P < 0.001.

To assess the clinical values of microstimulation in terms of therapeutic benefits to the patients, we compared the alterations in UPDRS/UDRS scores assessed during the trial stimulation and before the surgery (ΔUPDRS/ΔUDRS) as well as the therapeutic windows between the tracks with and without microstimulation. The therapeutic windows were 2.27 ± 0.12 V in the tracks with microstimulation (Stim tracks) and 1.93 ± 0.13 V in the tracks without microstimulation (Non-Stim tracks, *P* = 0.056, Figure 8A). The ΔUPDRS/ΔUDRS scores were 15.13 ± 1.24 in the Stim tracks, which were significantly higher than those of the Non-Stim tracks (10.69 ± 1.14, *P* = 0.010, Figure 8B). Besides this, we also compared the ΔUPDRS/ΔUDRS scores and the therapeutic windows between the tracks in which the SSB was identified vs. the ones that were not. The therapeutic windows were 2.31 ± 0.11 V in the SSB-identified tracks (SSB tracks) and 1.85 ± 0.14 V in the SSB-unidentified tracks (Non-SSB tracks). The ΔUPDRS/ΔUDRS scores were 15.46 ± 1.23 in the SSB tracks and 9.78 ± 0.97 in Non-SSB tracks. Both the therapeutic windows and the ΔUPDRS/ΔUDRS scores were higher in the SSB tracks than in the Non-SSB tracks (*P* = 0.014 and 0.001, respectively, Figures 8C,D). These results show that microstimulation is promoting better therapeutic effectiveness.

## Discussion

A previous study has suggested the great potential of microstimulation in localizing the ventral border of STN (15). However, the study only tests on four patients, without evidence from imaging techniques and clinical assessments confirming the improvement on the lead placement and clinical effectiveness using this method. Our study is an extension of the previous study to evaluate the feasibility of microstimulation using more sophisticated methods and neurophysiological data from 41 patients. Our results showed that, in most trajectories with microstimulation (84.4%), inhibition of neural activity could be induced at the top part of the SNr but not at the bottom of the STN. Such difference in responses to the microstimulation of STN and SNr can be used as a tool to facilitate the localization of the STN exit and the SNr entrance (*i*.*e*., the identification of SSB) during microelectrode recording, which helps to place the electrode tip inside or very close to the SSB, resulting in higher contact quantity inside the STN. Such placement of DBS leads is considered optimal for DBS surgery. In other words, our results demonstrated that microstimulation could be used to promote the identification of SSB and therefore contribute to better lead placement. This finding is supported by the wider therapeutic window and the greater decline in UPDRS/UDRS scores identified in tracks with microstimulation than those without. These results further indicate that microstimulation contributes to better therapeutic effectiveness.

Our results also showed some opposite scenarios, in which the microstimulation triggered inhibition in STN and failed to induce inhibition in SNr. Nevertheless, there were also a few units that acted differently to microstimulation in previous studies, whose percentage of outliers (1.4% for STN and 15.4% for SNr) was similar to ours (15, 22). Thus, our results are generally consistent with the previous findings (15). In the light of the close relationship between the SNr and the ventral border of the STN (see “INTRODUCTION”), it is possible that other factors acted as confounds, including misinterpretation of the real microelectrode location, slight slide of the recording electrode, alteration in neuronal activity, tiny movement of the cables that transfer the signal, *etc*. Thus, it is difficult to determine whether these outliers reflected the real characteristics of these nuclei in response to microstimulation.

The location of DBS electrodes relative to the STN is important to the therapeutic effects of DBS (as is discussed in the “INTRODUCTION”) (18). The key to the optimal placement of DBS electrodes is to cover the dorsolateral STN as much as possible (5, 21). Although the final placement of DBS leads is a clinical decision that depends on a myriad of factors, placing the electrode tip near the SSB is still recommended by most neurosurgeons to ensure better coverage of motor STN and fewer side effects (5, 11). During the surgery, it is often very difficult to determine where to stop in the STN because the signal near the ventral boundary of the STN is sometimes elusive (6, 11, 25). The gap between the STN and SNr can be very small that surgeons can misinterpret the SNr signal as the STN signal. In this case, microstimulation can be applied to test the different responses of these nuclei and contribute to the correct judgment on the real location of the microelectrode. The advantages of this method are as follows: (1) reliability: both our study and previous studies showed a high replicability of such phenomenon and a low percentage of outliers, and our results from the reconstruction of DBS leads, in turn, verified the judgment on the location of the SSB; (2) simplicity: this method is easy to apply, and the entire length of one trial of microstimulation takes no more than 10 s; and (3) safety: no abnormal scenario was induced or reported in our study. Therefore, we recommend that microstimulation can be conducted during routine microelectrode recording or only when the surgeons are doubtful of the microelectrode location.

### Limitations

The study that we presented here is primarily limited by the selection of trajectories. We only picked tracks that electrophysiologically displayed a typical STN signal and radiologically traveled through a good portion of the STN (shown by reconstruction). Another limitation of our study lies in the reconstruction method that we employed. Lead DBS software projects the DBS leads to standardized brain and atlas after normalization. Although studies have verified the accuracy of Lead DBS (23, 26), a tiny discordance between the computed nuclei and the real brain may exist. Therefore, a future study with a better design is needed to investigate the efficacy of microstimulation for guiding lead implantation.

## Conclusion

Our study tested the efficacy of a previous finding which has not been widely applied in DBS surgery: microstimulation can work as a tool to aid in targeting the ventral border of STN. Our results show that microstimulation can be used to promote the identification of the gap between the STN and SNr and thus can contribute to better lead placement.

## Data Availability Statement

The original contributions presented in the study are included in the article/supplementary material, further inquiries can be directed to the corresponding author/s.

## Ethics Statement

The studies involving human participants were reviewed and approved by Ethic Committee of Beijing Tiantan Hospital. The patients/participants provided their written informed consent to participate in this study.

## Author Contributions

AY, JZha, and LS conceived and designed the study. LS, SF, TY, QZ, YD, HZ, HL, and FM performed the study and collected data. JZhe, HF, and ZX investigated the data and performed statistical analyses. LS, HF, JZhe, and QZ wrote the first draft. JZhe, AY, and JZha revised the paper. All authors met the requirements for authorship. All authors believe the manuscript represents honest work and approved the submission and publication of the final version of the manuscript.

## Funding

This research was supported by National Nature Science Foundation of China (Grant Number 81701268, 81870888), the Young Scientist Program of Beijing Tiantan Hospital (Grant Number YSP201903), and the Capital Medical Development Research Fund (2018-2Z-1076).

## Conflict of Interest

The authors declare that the research was conducted in the absence of any commercial or financial relationships that could be construed as a potential conflict of interest.

## Publisher's Note

All claims expressed in this article are solely those of the authors and do not necessarily represent those of their affiliated organizations, or those of the publisher, the editors and the reviewers. Any product that may be evaluated in this article, or claim that may be made by its manufacturer, is not guaranteed or endorsed by the publisher.
